# Assessment of asthma and chronic obstructive pulmonary disorder in relation to reversibility, IgE, eosinophil, and neutrophil count in a University Teaching Hospital in South Delhi, India

**DOI:** 10.4103/0975-7406.72136

**Published:** 2010

**Authors:** Virender P. Singh Rathod, Prem Kapoor, K. K. Pillai, Razia Khanam

**Affiliations:** Department of Pharmacology, Faculty of Pharmacy, Majeedia Hospital, Jamia Hamdard University, New Delhi - 110 062, India

**Keywords:** Asthma, bronchodilator, chronic obstructive pulmonary disorder, eosinophil, immunoglobulin E, neutrophil

## Abstract

**Objectives:**

The physiological and clinical similarities between asthma and chronic obstructive pulmonary disorder (COPD) make their differentiation difficult. In the present study, we compared reversibility to bronchodilator, immunoglobulin E (IgE), blood eosinophil and neutrophil levels among asthma and COPD patients to differentiate these diseases.

**Materials and Methods:**

The study was carried on 20 asthmatics and 29 patients of COPD that reported to the outpatient and inpatient department in University Teaching Hospital, Jamia Hamdard, New Delhi, India. The parameters evaluated included pulmonary function (FEV_1_, FVC, and FEV_1_/FVC), IgE levels, and eosinophil and neutrophil count.

**Results:**

It was observed that reversibility was significantly higher in asthmatic patients, while irreversibility predominates in COPD patients. There was no significant difference in pre- and post-FEV_1_ and pre- and post-FVC and in their percentage predicted. However the percentage change in FEV_1_ significantly varies in asthma and COPD patients. No significant changes in neutrophil and eosinophil levels were observed in these patients. The serum IgE levels were found significantly higher in asthmatic patients.

**Conclusions:**

We conclude that reversibility in FEV_1_ levels or percentage change in FEV_1_ and serum IgE levels are promising lab parameter to distinguish these two conditions. However, further research is required to fully understand the role of neutrophil and eosinophil in the onset and development of asthma and COPD.

Bronchial asthma and chronic obstructive pulmonary disease (COPD) are two airway chronic diseases that exact an enormous toll on patients, healthcare providers, and the society. Asthma, where airway inflammatory component occupies a preponderant place is characterized by recurrent episodes of several symptoms such as breathlessness, wheezing, chest tightness, airway obstruction, and cough.[1] The essential alterations of asthma includes bronchospasm (hyper reactivity), edema and mucous hypersecretion (bronchial obstruction), etc. It is estimated that around 300 million people currently have asthma worldwide.[2] On the other hand, COPD is a disorder of progressive airflow limitation caused by chronic inflammation of the airways and lung parenchyma and associated with symptoms such as cough, sputum production, and dyspnoea. Smoking is the primary risk factor for the development of COPD. Worldwide, it ranks as the fourth leading cause of death, alongside HIV/AIDS. By the year 2020, COPD is predicted to become the third leading cause of death worldwide (exceeded only by heart disease and stroke).[3] Although both of them have similar characteristics such as the signs of coughing and wheezing, they are two distinct conditions in terms of disease onset, frequency of symptoms, and reversibility of airway obstruction. There has been a substantial increase in the prevalence of both diseases that has lead to sizable concerns being expressed from national and international healthcare authorities.

Exacerbations of asthma often have identifiable triggers such as allergens, cold air, or exercise. However, exacerbations in COPD patients are commonly caused by respiratory tract infections. Both these diseases were once considered to be at the opposite ends of the spectrum of airway disease, where asthma was thought to be highly responsive to treatment and essentially reversible; COPD was characterized by fixed airway narrowing and unresponsive to the treatment. The currently accepted definitions still emphasize these features, but there may be significant overlap between the two diseases. Today it is recognized that these two conditions are distinct but their clinical features overlap and it is often difficult to differentiate, clinically diagnose, and classify the two disorders.[4,5]

Asthma and COPD are highly complex, many different inflammatory cells and multiple mediators with complex acute and chronic effects on the airways are part of the syndromes. Many different inflammatory cells are involved in asthma, although the precise role of each cell type is not yet certain. The inflammation in asthmatics airways differs strikingly from that observed in COPD, where there is a predominance of macrophages, cytotoxic (CD8+), lymphocytes, and neutrophils, although both these common diseases may coexist in some patients. Hence, the development of markers by which these two diseases can be separated out is imperative.

The criteria for diagnosis of asthma and COPD includes patients medical history, physical examination, spirometry especially pre- and post-FEV_1_, chest radiograph, differential diagnosis, etc. The present study was designed to compare asthma and COPD in relation to reversibility, IgE, eosinophils, and neutrophils count.

## Materials and Methods

### Patient

Adult patients suffering from asthma or COPD of either sex attending the outpatient and inpatient department of University Teaching Hospital, Jamia Hamdard, New Delhi, India, were included in the study. Out of a total of 1348 patients visited, 20 patients of asthma and 29 patients of COPD were selected, on the basis of history and clinical examinations. Asthma was diagnosed by the history of recurrent episodes of breathlessness and wheezing with or without cough and phlegm with seasonal and diurnal variations and identifiable triggering factors. COPD was diagnosed on the basis of history of smoking, cough with expectoration occurring for at least 3 months in a year for 2 years or more accompanied by progressive dyspnoea on exertion.

Patients who had taken an inhaled bronchodilator within the past 12 hours or an oral bronchodilator within the past 24 hours were excluded. Patients with coronary artery disease, systemic hypertension, previous history of pulmonary tuberculosis, concurrent pulmonary disease, children, pregnant, and breast feeding females, patients on long-term antihistaminics, and patients unable to comply were also excluded. The study was carried out in accordance with the basic principles defined in ‘Good Clinical Practice (GCP). It was approved by the Jamia Hamdard Institutional Review Board (JH-IRB). Informed written consent was obtained from each patient.

The characteristics of patients selected for the study are given in Table 1.

**Table 1:** Characteristics of the patients of asthma and COPD groups

Parameters	Asthma	COPD
No of subjects	20	29
Male/female ratio	0.67	6.25
Average age	33.15 ± 3.33	56.344 ± 1.79
Average weight	54.15 ± 2.64	52 ± 1.9
Smokers/nonsmokers ratio	0.05	2.63

### Methods

Spirometry: It was performed with subjects in sitting position and highest value of forced expiratory volume in 1 sec (FEV_1_) and forced vital capacity (FVC) were obtained. Three acceptable and at least two reproducible curves were obtained in each subject. The baseline spirometry was then performed. Then salbutamol was administered by metered-dose inhaler. It was given by keeping it about 3 cm in front of open mouth. It was repeated 20 min after administration of salbutamol. Reversibility was calculated in asthma and COPD patient groups.

Biochemical analysis: Blood samples (5.0) were collected from each subject by venipuncture. They were analyzed for absolute eosinophil count estimation on the same day of the sampling schedule at pathology laboratory, Majeedia hospital. Absolute eosinophil count (AEC) was made from the total leukocyte count (TLC) and differential leukocyte count (DLC) by indirect method and by the direct method using light microscopy. Serum was separated and stored frozen in aliquots (0.5 ml) until analyzed for immunoglobulin E (IgE) levels. Microparticle Enzyme Immunoassay method (Calboitech, U.S.A) was used for the quantitative measurement of serum IgE.

### Statistical analysis

Graphpad software was used for analyzing the data for unpaired ‘*t*’ test and mean ± SEM (wherever applicable). A value of *P* <0.05 was considered significant.

## Results

### Comparison of pulmonary functions in asthma and COPD patients

Table 2 shows the comparison of pulmonary functions in asthma and COPD groups. Asthmatic patients showed high reversibility while irreversibility in COPD patients was higher. The reversible/irreversible ratio in asthma (15/5) is 3, whereas in COPD (4/25) is 0.16. There was no significant difference in pre- and post-FEV_1_ and in also pre- and post-FVC and their percentage predicted in asthma and COPD Patients. However, percentage change in FEV_1_ significantly varies in asthma and COPD patients (*P* <0.0001, unpaired *t* test).

**Table 2:** Comparison of pulmonary functions in asthma and COPD groups

Parameters	Asthma	COPD
No of subjects	20	29
Reversible/irreversible ratio	3	0.16
Pre-FEV_1_ (L)	1.517 ± 0.13	1.3348 ± 0.08
Post-FEV_1_ (L)	1.868 ± 0.15	1.3955 ± 0.08
Pre-FVC (L)	1.841 ± 0.11	1.6337 ± 0.08
Post-FVC (L)	2.14 ± 0.12	1.73 ± 0.08
Pre-FEV_1_ (% predicted)	59.53 ± 2.99	52.766 ± 2.32
Post-FEV_1_ (% predicted)	66.49 ± 3.62	55.693 ± 2.55
Pre-FVC (% predicted)	56.25 ± 1.74	51.165 ± 1.81
Post-FVC (% predicted)	65.35± 1.99	54.406 ± 2.11
% Change FEV_1_	24.217 ± 3.83*	4.74 ± 0.89

FEV_1_, forced expiratory volume in 1 sec. FVC, forced vital capacity.

* *P*<0.0001, asthma vs COPD. Unpaired *t* test (two-tail *P* value).

### Comparison of blood cells in asthma and COPD patients

Table 3 depicts the levels of blood cells in asthma and COPD group. The TLC was higher in COPD patients, though nonsignificant. Eosinophil count was slightly higher in asthma patients and the neutrophil count was higher in COPD patients, but the difference was nonsignificant.

**Table 3:** Comparison of blood cells in asthma and COPD patients

Parameters	Asthma	COPD	Normal range
No of subjects	20	24	
TLC (/cmm)	8759 ± 640.99	10374.58 ± 884.90	4000–11000
Eosinophils(/cmm)	663.4 ± 64.03	588.49 ± 4.57	Up to 660
Neutrophils(/cmm)	5026.85 ± 383.18	7021.27 ± 850.14	1800–7700

TLC, total leukocyte count. *P*>0.05, results not significant. Unpaired *t* test (two-tail *P* value).

### Comparison of IgE levels in asthma and COPD patients

Table 4 illustrates the levels of IgE in asthma and COPD patients. Both the groups showed higher IgE level than the normal range, but in asthma patients it was significantly higher as compared to COPD group (*P* <0.0001, unpaired *t* test).

**Table 4:** Comparison of IgE levels in asthma and COPD patients

Parameters	Asthma	COPD
No. of subjects	13	15
IgE level (IU/ml)	490.60 ± 27.93*	147.02 ± 29.26

IgE, immunoglobulin E. Results are expressed as mean ± SEM.

* *P*< 0.0001 (unpaired *t* test, two tail *P* value).

## Discussion

Our study was designed with the objective whether airways reversibility, number of eosinophils, neutrophils, and IgE levels in asthma and COPD patients can be used as a marker to distinguish these diseases. It was observed that asthma patients showed high airway reversibility while COPD patients showed high airway irreversibility. These results are in agreement with other clinical studies showing the same pattern of observation.[6–8] The assessment of a bronchodilator response is a routine procedure both in pulmonary medicine and in research. These responses are commonly used as a basis for classification of disease and choice of treatment by clinicians and as an inclusion criterion for research studies. However, there is as yet no consensus as to which index best expresses bronchodilator response, or even as to what cut-off value would indicate a positive bronchodilator response. An increase in FEV_1_ greater than a threshold value is assumed to represent reversibility of airways obstruction. The criteria suggested for positive test of reversibility range from 12% to 20% increase in FEV_1_, over the baseline value.[9,10] A change in FEV_1_≥9.10 of the predicted FEV_1_ (European Respiratory Society)[11] and change in FEV_1_≥ 12% and 0.2l over the baseline (American Thoracic Society)[12] has been specified. On the contrary, few studies have shown that these thresholds have little value in separating patients with bronchial asthma and COPD.[13–15] It is also reported that patients with COPD respond to bronchodilator more often by an increase in the FVC than in FEV_1_, hence consideration of changes in both FEV_1_ and FVC to evaluate bronchodilator responsiveness has been emphasized. We have evaluated pre- and post-FEV_1_ and FVC in both the patients and found that FVC increased from the baseline value after bronchodilator dosage in asthmatic as well as in COPD patients. FEV_1_ also increased from the baseline after bronchodilator treatment in both the patients, but the increase was more marked in asthmatic patients. In addition, difference in percentage change in FEV_1_ was statistically significant. Asthmatics showed more than 12% increase in FEV_1_ after bronchodilator. On the other hand, bronchodilator response in COPD patients was limited. Thus on the basis of our observation, it was found that bronchodilator reversibility (% FEV_1_) is a significant parameter to separate asthma from COPD.

In our study, the TLC in asthma patients was in normal range, but slightly higher in COPD patients. Deference in TLC level in both groups was also not significant. It is well-established fact that cigarette smoking is associated with an elevated leukocyte count in the peripheral blood and lungs.[16] Most of the COPD patients were smokers in our study. Slight increase in TLC in COPD patients may be due to smoking. Eosinophil count in COPD patients was in normal range, though it was higher in asthma patients but not statistically significant difference in eosinophil count was observed between these groups. Eosinophilic inflammations in asthma are well known,[17] but are also reported in the airways of some COPD patients and have a correlation with severity of airflow obstruction.

Increased neutrophil count in COPD patients was also observed, as compared to asthmatic group. It is well known that neutrophils play a crucial role in the pathophysiology of COPD, as they release multiple mediators and tissue degrading enzymes such as elastases that orchestrate tissue destruction and chronic inflammation.[18] Neutrophil numbers are also increased in bronchoalveolar lavage fluids from cigarette smokers.[19] In our study, most of the patients of COPD group were smokers. Hence its role in airways injury including COPD is a matter of speculation, and advanced as well as more targeted studies can provide a relation between neutrophils and lung injury. It has also been reported recently that COPD is associated with sputum neutrophilia and asthma with sputum eosinophilia.[20]

We have also investigated the levels of IgE with asthma and COPD patients. Since IgE molecules are known to have a role in the pathogenesis of both the diseases. In our study, we observed a significant increase in serum IgE levels of asthma patients in comparison to COPD patients. Recently, Mahajan *et al*. have also observed a significant increase in serum IgE levels of atopic asthmatics in comparison with nonatopic asthmatics, COPD patients, and healthy individuals.[21] Sin has shown that asthmatics also had higher serum IgE level than COPD patients, although the difference was not significant.[8] In another study, a significantly raised serum total IgE level has been detected in asthmatic patients.[7] In our study, IgE level of COPD patients was also higher than normal range, which could be due to the degree of tobacco smoking[22,23] or local production of IgE in the bronchial mucosa.[24]

The differential diagnosis of bronchial asthma and COPD is usually made on the basis of clinical history, physical examination, and the PFT. However, in some cases, these parameters fail to establish conclusively the diagnosis of these diseases. To conclude, our study highlighted a statistically significant difference in the reversibility of FEV_1_ of the magnitude of 12% in case of bronchial asthma and increased IgE levels as compared to COPD patients. Hence, it suggests that reversibility in FEV_1_ level and serum IgE levels are the promising lab parameters to distinguish the conditions. Since, the study was carried out over a 4-month period, and seasonal variations in disease pattern were not considered, further studies involving more number of patients in different hospitals and the influence of inflammatory mediators on the two diseases are further warranted.
